# Natural selection of protein structural and functional properties: a single nucleotide polymorphism perspective

**DOI:** 10.1186/gb-2008-9-4-r69

**Published:** 2008-04-08

**Authors:** Jinfeng Liu, Yan Zhang, Xingye Lei, Zemin Zhang

**Affiliations:** 1 Department of Bioinformatics, Genentech Inc., 1 DNA Way, South San Francisco, CA 94080, USA; 2 Department of Biostatistics, Genentech Inc., 1 DNA Way, South San Francisco, CA 94080, USA

## Abstract

**Background:**

The rates of molecular evolution for protein-coding genes depend on the stringency of functional or structural constraints. The Ka/Ks ratio has been commonly used as an indicator of selective constraints and is typically calculated from interspecies alignments. Recent accumulation of single nucleotide polymorphism (SNP) data has enabled the derivation of Ka/Ks ratios for polymorphism (SNP A/S ratios).

**Results:**

Using data from the dbSNP database, we conducted the first large-scale survey of SNP A/S ratios for different structural and functional properties. We confirmed that the SNP A/S ratio is largely correlated with Ka/Ks for divergence. We observed stronger selective constraints for proteins that have high mRNA expression levels or broad expression patterns, have no paralogs, arose earlier in evolution, have natively disordered regions, are located in cytoplasm and nucleus, or are related to human diseases. On the residue level, we found higher degrees of variation for residues that are exposed to solvent, are in a loop conformation, natively disordered regions or low complexity regions, or are in the signal peptides of secreted proteins. Our analysis also revealed that histones and protein kinases are among the protein families that are under the strongest selective constraints, whereas olfactory and taste receptors are among the most variable groups.

**Conclusion:**

Our study suggests that the SNP A/S ratio is a robust measure for selective constraints. The correlations between SNP A/S ratios and other variables provide valuable insights into the natural selection of various structural or functional properties, particularly for human-specific genes and constraints within the human lineage.

## Abstract 2

A large-scale survey using single nucleotide polymorphism data from dbSNP provides insights into the evolutionary selection constraints on human proteins of different structural and functional categories.

## Background

It is well established that there are tremendous variations in rates of evolution among protein-coding genes. A central problem in molecular evolution is to identify factors that determine the rate of protein evolution. One widely accepted principle is that a major force governing the rate of amino acid substitution is the stringency of functional or structural constraints. Proteins with rigorous functional or structural requirements are subject to strong purifying (negative) selective pressure, resulting in smaller numbers of amino acid changes. Therefore, these proteins tend to evolve slower than proteins with weaker constraints. A classic measure for selective pressure on protein-coding genes is the Ka/Ks ratio [1], that is, the ratio of non-synonymous (amino acid changing) substitutions per non-synonymous site to synonymous (silent) substitutions per synonymous site. The assumption is that synonymous sites are subject to only background nucleotide mutation, whereas non-synonymous sites are subject to both background mutation and amino acid selective pressure. Thus, the ratio of the observed non-synonymous mutation rate (Ka) to the synonymous mutation rate (Ks) can be utilized as an estimate of the selective pressure, where Ka/Ks « 1 suggests that most amino acid substitutions have been eliminated by selection, that is, strong purifying selection. Ka/Ks ratios for protein-coding genes are generally derived from inter-species sequence alignments and different evolution models have been developed to accurately estimate the ratios [2]. There have been many studies using Ka/Ks ratios to measure evolutionary constraints among different classes of proteins. For example, it has been suggested that essential genes in bacteria evolve slower than non-essential genes [3], that house-keeping genes are under stronger selective constraints than tissue-specific genes [4], and that secreted proteins are under less purifying selection based on Ka/Ks ratios from human-mouse sequence alignments [5].

In the past few years, advances in sequencing technology have led to a rapid accumulation of DNA variation data for human populations, including copy number variations and single nucleotide polymorphisms (SNPs). Currently, the dbSNP database [6] at the National Center of Biotechnology Information (NCBI) catalogues about 12 million human SNPs, close to half of which are validated. It has also been shown by several independent sequencing studies that dbSNP has high coverage of frequent SNPs [7,8]. The vast amount of SNP data can not only shed light on the variation in disease susceptibility and drug response among human populations, but also help us understand molecular evolution. In particular, these SNP data have provided us with another way of measuring evolutionary constraints, based on a prediction of the neutral theory of molecular evolution that A/S ratios should be highly correlated between intra-species polymorphism and inter-species divergence [9]. In fact, SNP A/S ratios (also referred to as Ka/Ks ratios for polymorphisms) have been calculated to determine whether there is frequent positive selection on the human genome [10,11], and they have been compared with Ka/Ks for human-chimpanzee divergence [12]. However, it is not clear whether SNP A/S ratios are closely correlated with Ka/Ks in practice given the current volume of SNP data, and there have not been any large-scale studies of selective constraints on protein structural and functional properties using SNP data.

In the present study, we conducted a large-scale survey of SNP A/S ratios using SNP data from dbSNP. We first confirmed that the SNP A/S ratio is a good measure for selective pressure by showing its correlation with Ka/Ks from inter-species alignments and protein alignment conservation. We then obtained a variety of structural and functional properties from either database annotations or computational prediction methods and analyzed SNP A/S ratios for different classes of proteins and residues in an attempt to study the natural selection of these properties from the SNP perspective. Our comprehensive analysis provides: valuable insight into some features that have not been examined previously; independent confirmation of some previously established results; and additional data for areas where previous studies have had contradictory findings.

## Results

We collected 13,686 human genes that have at least one validated coding SNP according to dbSNP. The analysis was limited to validated SNPs to ensure data quality. Overall, 45,538 coding-region SNPs and 1,529,119 intronic SNPs were identified in these genes, corresponding to SNP densities of 2.0 and 2.4 SNPs, respectively, per 1,000 nucleotides. The number of non-synonymous coding SNPs per non-synonymous site (A) is 0.00123, the number of synonymous coding SNPs per synonymous site (S) is 0.00439, and the A/S ratio is 0.28. The values of A and S are both two times more than what have been reported in a small study [11], but the A/S ratio is similar.

### SNP A/S ratio as a measure for selective constraints

**Figure 1:**
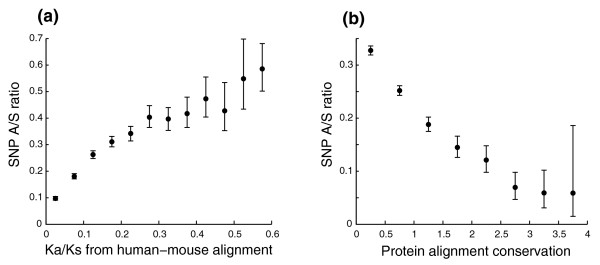
The SNP A/S ratio is a good measure for evolutionary constraints. Error bars represent 95th percentile confidence intervals from bootstrap resampling. **(a) **SNP A/S ratios correlate with Ka/Ks ratios from human-mouse alignments. Proteins were grouped into bins of equal intervals (interval = 0.05) according to their Ka/Ks ratios, and the SNP A/S ratio was calculated for each bin. **(b) **SNP A/S ratios correlate negatively with residue conservation scores from protein sequence alignments. All residues were grouped into bins of equal intervals (interval = 0.5) according to their position specific alignment information taken from PSI-BLAST alignment profiles, and the SNP A/S ratio was obtained for each bin.

To assess whether SNP A/S ratios from the current large-scale SNP data set provide a good measure for selective constraints, we first compared them with Ka/Ks ratios derived from inter-species alignments. We collected 9,759 human proteins with both validated coding-region SNPs and available human-mouse Ka/Ks data from Ensemble [13], binned them by their Ka/Ks values, and measured the SNP A/S ratios for each group. There is a strong positive correlation between these two measure (Figure 1a; Kendall's rank correlation [14] τ = 0.50, *p*-value < 1e-04), which is in agreement with the neutral theory of molecular evolution. Analysis of data from chimpanzee and Old World monkey (*Macaca mulatta*) led to similar conclusions, although the Ka/Ks values may need to be corrected to subtract the contribution of SNPs due to relatively short evolutionary distance.

We next investigated whether the conservation in protein sequences correlates with the SNP A/S ratio under the assumption that both the conservation at the protein sequence level and the SNP A/S ratio at the nucleotide level are indications for selective constraints. Using the position-specific alignment entropy (a measure for conservation) from PSI-BLAST profiles [15], we calculated A/S ratios for residues with different conservation scores. We indeed observed a monotonic decrease of the A/S ratio with an increase in protein sequence conservation (Figure 1b). The residues with the conservation range of 0-0.5 have a ratio of 0.33, while those having conservation scores bigger than 3.5 have an A/S ratio of 0.06.

### SNP A/S ratios for protein features

Many studies have been published addressing the correlation between evolutionary constraints and other variables, most of which were based on relatively small data sets. Having established the SNP A/S ratio as a good measure for selective constraints, we attempted to use the large-scale human SNP data set to revisit some of the features in the earlier studies, and also to investigate several protein properties that had not been examined before.

### Selective constraints and mRNA expression

**Figure 2:**
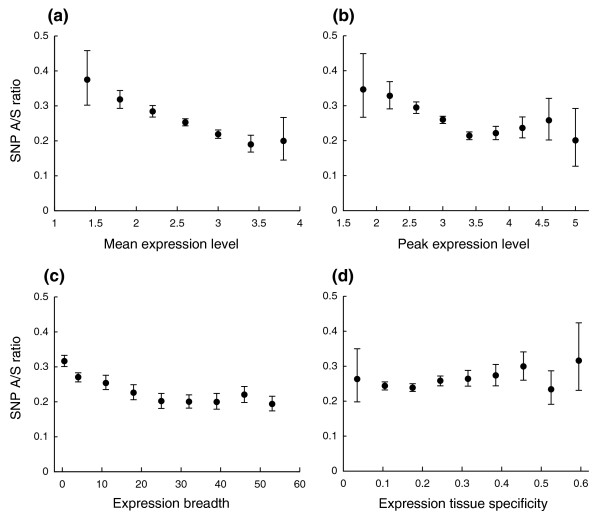
Correlation between SNP A/S ratios and expression parameters. Genes were grouped into bins of roughly nine equal intervals according to several expression measurements from a microarray experiment, and the SNP A/S ratio was obtained for each bin. Error bars represent 95th percentile confidence intervals from bootstrap resampling. **(a) **Negative correlation between SNP A/S ratios and mean mRNA expression levels. **(b) **Negative correlation between SNP A/S ratios and peak mRNA expression levels. **(c) **Negative correlation between SNP A/S ratios and expression breadth. **(d) **No correlation between SNP A/S ratios and expression tissue specificity.

Until a few years ago, the prevalent theory in molecular evolution was that evolutionary rate is largely dependent on structural and functional constraints. Recently, increasingly more evidence suggests that there is a strong correlation between evolutionary rate and gene expression. It has been observed that highly expressed genes evolve slowly in bacteria [16], yeast [17], and mammals [18]. In yeast, it has been shown by principal component regression that the number of translation events is the dominant determinant of evolutionary rate among several other functional attributes [19], leading to the increasingly popular 'translational robustness' hypothesis [20]. However, a later study suggested that the dominant effect may result from the noise in biological data that confounded the analysis [21]. Studies of human mRNA expression data showed that the breadth of expression (that is, the number of tissues in which a gene is expressed) also correlates with evolutionary rate [22,23]; it is still debatable whether the breadth or the rate of expression is the stronger predictor [18]. We obtained mRNA expression data for 10,885 genes in our data set that are available from a published microarray experiment (Gene Expression Atlas) [24] and investigated the correlation between selective constraints and four gene expression parameters examined previously: peak expression level, mean expression level, expression breadth, and tissue specificity. Overall, this set of genes with available mRNA expression data has an SNP A/S ratio of 0.25, lower than that of our entire data set (0.28). We indeed observed that highly expressed genes tend to have low A/S ratios (Figure 2a,b): both mean and peak expression rate negatively correlate with the SNP A/S ratio (τ = -0.178 and -0.160, respectively; Table S1 in Additional data file 1). Genes with the lowest mean expression levels have an A/S ratio of 0.38, about twice as high as the ratio in the highest expression group (Figure 2a). The SNP A/S ratio also correlates well with the breadth of expression (Figure 2c; τ = -0.213, *p*-value < 1e-04), but only marginally with tissue specificity (Figure 2d; τ = 0.047, *p*-value = 0.003). Since these four expression parameters correlate strongly with each other, we carried out partial correlation analysis [14] to identify the stronger predictors for evolutionary rates. The correlation between tissue specificity and the A/S ratio disappeared entirely after controlling for mean expression level (τ = 0.0107, *p*-value = 0.499; Table S1 in Additional data file 1) or expression breadth (τ = 0.0084, *p*-value = 0.596; Table S1 in Additional data file 1). Expression breadth and mean expression level both remain significantly correlated with the A/S ratio when controlling one for the other (τ = -0.096 and -0.064, *p*-values < 1e-04 and 7e-04, respectively; Table S1 in Additional data file 1). Peak expression level is highly correlated with mean expression level and its partial correlation patterns largely resemble those of mean expression level. It has recently been recognized that it is critical to control for expression when studying the statistical relevance of other variables as predictors for evolutionary rates, since many previously reported correlations became insignificant after this control. As expression breadth appeared to have the strongest correlation with the SNP A/S ratio in our data set among the four parameters, we chose to control for it in the following correlation analysis between selective constraints and other variables. The results did not change qualitatively when controlling for mean expression level instead.

### SNP A/S ratio and evolutionary variables

**Figure 3:**
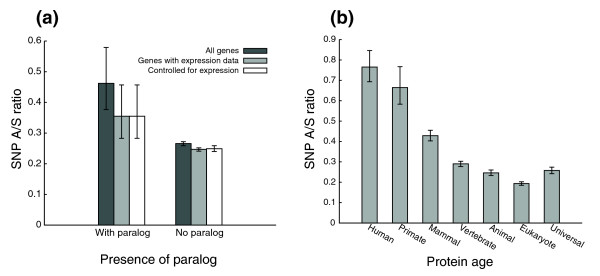
SNP A/S ratios and evolutionary variables. **(a) **Proteins with paralogs (167 proteins) are under weaker selective pressure than proteins without paralogs (12,460 proteins). The 95th percentile confidence intervals of the A/S ratio are [0.38, 0.58] for proteins with paralogs, and [0.26, 0.27] for proteins without paralogs (dark gray bars). To control for expression breadth, the subset of proteins with mRNA expression data were analyzed (65 proteins with paralogs and 10,612 without, light gray bars) and Monte Carlo samplings were performed so that the two groups had the same distribution of expression breadth. The differences in A/S ratios are significant both before (light gray bars) and after (white bars) controlling for expression. **(b) **Proteins that arose early in evolution are subject to stronger evolutionary constraints.

Consistent with the hypothesis that gene duplications are an important source of new protein function, it has been observed that duplicated genes evolve under weaker purifying selection than unduplicated ones [25,26]. We collected 12,460 human genes without paralogs and 167 genes with paralogs according to the HomoloGene database [27,28], and found that the A/S ratio is markedly higher for genes with paralogs (0.46 versus 0.27, *p*-value < 1e-04; Figure 3a, dark gray bars). To control for expression breadth, we analyzed the subset of genes with mRNA expression data from the Gene Expression Atlas [24]. The two groups of genes do not differ in their distribution of expression breadth (Kolmogorov-Smirnov test, *p*-value = 0.507). The difference in the A/S ratio did not change significantly when the expression breadth was controlled by Monte Carlo sampling (Figure 3a, light gray bars and white bars). We then examined whether the higher rate could be solely explained by additional copies of paralogs while keeping one copy stable. When we selected the fastest evolving genes from each homology group, they have an A/S ratio of 0.55 compared with 0.36 for the batch of the slowest-evolving genes from each homology group. Both numbers are higher than the A/S ratio for genes without paralogs (0.27), suggesting that both duplicated copies are evolving faster than unduplicated genes. The much bigger variation in the with-paralog group (95th percentile confidence interval = [0.38, 0.58]) reflects the small number of genes in that particular group.

To determine whether the SNP A/S ratio correlates with the age of proteins, we classified each protein into one of seven age groups according to their most ancient homologs. It appears that young proteins (for example, those found in human or primates only) have the highest A/S ratios (0.76 for human and 0.66 for primates), whereas proteins traceable to all animals or other eukaryotes have much lower ratios of about 0.25 (Figure 3b). This is consistent with a previous finding that proteins that arose earlier in evolution tend to have a larger proportion of sites subjected to negative selection [29], although there was some debate about whether the observation was an artifact resulting from the inability of BLAST to detect homology for the fastest-evolving genes [30,31]. We examined the functions of proteins in each group by their Gene Ontology (GO) [32] annotation of biological process. The human-specific group is the least well annotated, with only 6% having GO annotation compared with 62% overall and 84% for proteins conserved in both eukaryotes and prokaryotes (the 'universal' group). Among the proteins with GO annotation of biological process, we observed the enrichment of 'epidermis development', 'defense response to bacterium', and 'spermatogenesis' in the human and primate groups, whereas 'amino acid metabolic process', 'glycolysis', and 'fatty acid metabolic process' are overrepresented in the 'universal' group.

### SNP A/S ratios and sequence/structure variables

**Figure 4:**
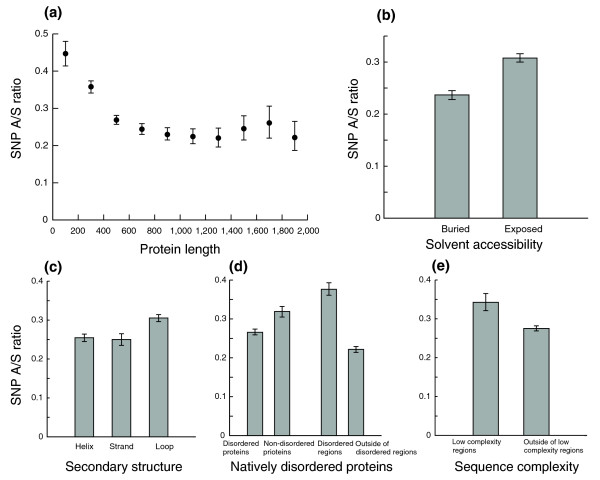
Evolutionary constraints on protein sequence and structure features. Error bars represent 95th percentile confidence intervals from bootstrap resampling. **(a) **For proteins shorter than 500 residues, short proteins have high A/S ratios. **(b) **Buried residues are under stronger selection. The 95th percentile confidence intervals of the A/S ratio are [0.23, 0.25] for buried residues, and [0.30, 0.32] for exposed residues. **(c) **Loop residues have relaxed evolutionary constraints. The 95th percentile confidence intervals of the A/S ratio are [0.25, 0.26] for residues in alpha-helices, [0.24, 0.27] for residues in beta-strands, and [0.30, 0.32] for residues in loops. **(d) **Proteins with disordered regions are more conserved, while disordered residues are under lower selective pressure. **(e) **Residues in low complexity regions evolve faster.

As an example of the many conflicting reports in the literature about correlations with evolutionary rates, for a variable as simple as protein length, it was shown that there was positive correlation [33], negative correlation [34,35], or no correlation [36]. In addition, there was a study based on protein sequence alignments that showed that less conserved proteins are shorter than more conserved ones on average [37]. In our data set, we observed a negative correlation between protein length and SNP A/S ratio (Kendall's τ = -0.137, *p*-value < 1e-04). The correlation did not change upon controlling for expression breadth. Our analysis also showed that this correlation is only prominent for proteins shorter than 500 residues, and disappears for longer proteins (Figure 4a).

Solvent accessibility measures the degree of an amino acid residue's exposure to the surrounding solvent. There have been a number of studies about the effect of mutations on solvent accessibility and its implication in human diseases; most of them were based on relatively small collections of SNPs in known protein structures. The general consensus was that buried residues are less likely to vary and their mutations are more likely to cause disease [38,39]. We obtained solvent accessibility predictions for all proteins in our dataset using PROFacc [40], and compared the SNP A/S ratios. Exposed residues have an A/S ratio of 0.31, significantly higher than that of 0.24 for the buried residues (Figure 4b). The *p*-value for this difference is smaller than 1e-04 according to bootstrap analysis. Similar results were obtained when using three-state prediction (buried, intermediate, and exposed) or numeric relative accessibility values. This underscores higher selective constraints on buried residues, possibly due to their importance in maintaining protein stability.

We also investigated selective constraints upon different protein structure conformations. We first grouped all residues into different secondary structure conformations (alpha-helix, beta-strand, or loop) according to predictions by PSIPRED [41]. Significantly higher A/S ratios were observed for residues in the loop conformation (Figure 4c), suggesting relaxed selective pressure on these residues. There is no difference between residues in alpha-helices and beta-strands. We next examined natively disordered proteins, a class of structurally flexible proteins that have recently gained traction because of their potential important roles in dynamic molecular recognition of macromolecules [42]. It has been estimated that one-third of eukaryotic proteins contains disordered regions [43], and that they are more likely to be involved in regulatory functions and protein-protein interactions [44,45]. We obtained disorder predictions using DISOPRED2 [43] and retained only the disordered regions longer than 30 residues. Interestingly, while proteins with disordered regions have a lower A/S ratio (Figure 4d; Figure S2b in Additional data file 1), the residues in disordered regions have a much higher A/S ratio than other residues (0.38 versus 0.22; Figure 4d). This seems to suggest that disordered proteins as a class are under stronger selective pressure, but the disordered residues are allowed to evolve much faster to explore different ways to interact with other molecules. Since disordered regions are often characterized by low sequence complexity [42,44], we also examined the selective constraints on low complexity regions as defined by SEG [46]. Not surprisingly, low complexity regions have a higher A/S ratio, but the profile is different from that of the disordered regions (Figure 4e), confirming that disorder and low complexity are related but different sequence features.

### SNP A/S ratios and protein subcellular localization

**Figure 5:**
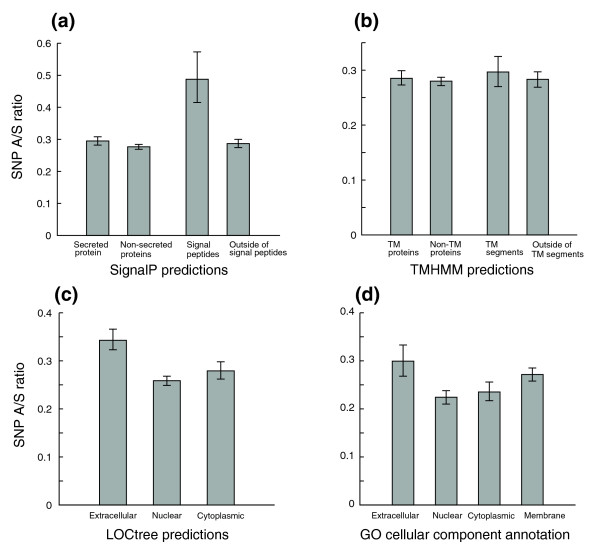
Selective pressures on protein subcellular localization. Error bars represent 95th percentile confidence intervals from bootstrap resampling. **(a) **Analysis of SignalP predictions suggests that while there is no significant difference in selective pressure between secreted and non-secreted proteins, residues within signal peptides are evolving faster. **(b) **TMHMM predictions show no difference in A/S ratios between membrane proteins and non-membrane proteins, transmembrane segments and non-transmembrane segments. **(c) **LOCtree predictions of protein subcellular localization indicate extracellular proteins (1,587 proteins) are under more relaxed selective pressure than cytoplasmic proteins (2,105) and nuclear proteins (5,431). **(d) **GO cellular component annotations suggest extracellular proteins (522 proteins) are under more relaxed selective pressure than cytoplasmic proteins (1,030) and nuclear proteins (1,961), while membrane proteins (2,715) fall in between. The 95th percentile confidence intervals of the A/S ratio are [0.27, 0.33] for extracellular proteins, [0.21, 0.24] for nuclear proteins, [0.22, 0.26] for cytoplasmic proteins, and [0.26, 0.29] for membrane proteins.

Subcellular localization is an important aspect of protein function. There have been conflicting reports about the correlation between protein subcellular localization and evolutionary rate. While a previous survey of human SNPs in 2002 did not find a significant correlation of selective pressure against deleterious non-synonymous SNPs with localization [47], a more recent study of mammalian sequences found that secreted proteins evolve much faster than cytoplasmic proteins (Ka/Ks 0.27 versus 0.12), and that membrane segments are under higher selective pressure than non-membrane segments (0.07 versus 0.15) [48]. We attempted to address this issue by examining A/S ratios from several subcellular localization assignment methods. When we divide our data set into 3,064 secreted proteins and 10,622 non-secreted proteins according to SignalP [49] predictions, there is a small and insignificant difference between these two classes, but the residues within the signal peptides appear under much less selective pressure (A/S ratios of 0.42 versus 0.29; Figure 5a). Interestingly, when only the subset of genes that have mRNA expression data was examined (both before and after controlling for expression), secreted proteins had significantly higher A/S ratios than non-secreted proteins (*p*-value < 1e-04; Figure S3a in Additional data file 1). There is no difference between membrane proteins and non-membrane proteins, membrane segments and non-membrane segments according to TMHMM [50] predictions (Figure 5b; Figure S3b in Additional data file 1). We also obtained predictions of subcellular localizations for non-membrane proteins by LOCtree [51], a hierarchical prediction system mimicking cellular sorting mechanisms. Predicted extracellular proteins have an A/S ratio of 0.34 on average, significantly higher than nuclear and cytoplasmic proteins (Figure 5c). Lastly, we examined A/S ratios of 6,228 proteins that have unambiguous GO cellular component assignments. We observed the same trend as for the LOCtree predictions, although the absolute numbers are slightly lower (Figure 5d). This may be explained by the fact that more conserved proteins are more likely to get GO annotation through sequence homology. The selective constraints acted upon membrane proteins seem to fall between the extracellular and cytoplasmic proteins according to the GO annotations (Figure 5d). The results from both LOCtree predictions and GO annotation did not change qualitatively when controlling for expression breadth (Figure S3c,d in Additional data file 1). Overall, our analysis suggests that extracellular proteins are indeed under more relaxed selection than cytoplasmic and nuclear proteins, but the difference is not as dramatic as previously reported. The absence of difference between membrane and non-membrane proteins according to TMHMM predictions may result from the lack of distinction between the extracellular and cytoplasmic/nuclear proteins.

### Selective constraints on functional classes and protein families

**Figure 6:**
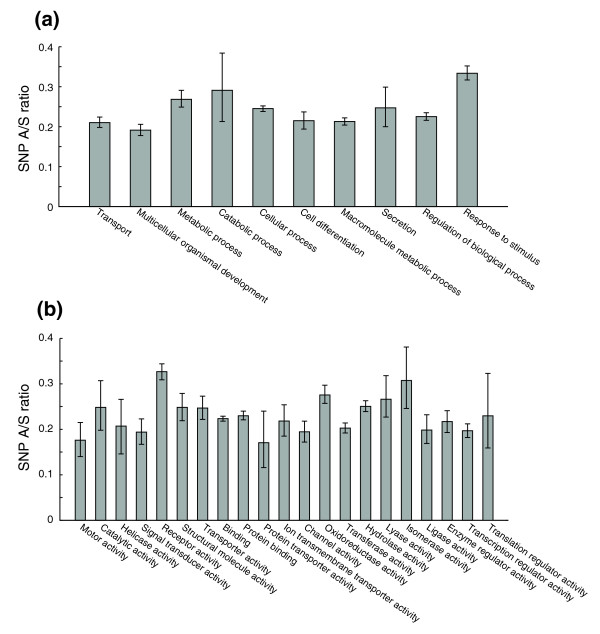
Evolutionary constraints on protein functional categories. Error bars represent 95th percentile confidence intervals from bootstrap resampling. GO annotations were extracted for each protein, and the GO terms were mapped to high level GOA slim terms for **(a) **biological process and **(b) **molecular function. SNP A/S ratios were then calculated for each group.

We next studied the variation in SNP distribution of functional categories based on GO annotations. A/S ratios were calculated for 176 GO biological process categories and 152 molecular function categories that have at least 20 genes in our data set. As expected, there are dramatic differences in selective constraints among different categories: A/S ratios range from 0.72 for 'sensory perception of smell' to 0.07 for 'protein kinase C activation' (Table 1). We compared our results with a comparative genomic study of human and chimpanzee [12]. Seven of the top ten categories with highest divergence rates between human and chimpanzee are not present in our entire set of 176 categories due to differences in gene sets and the availability of SNP data. Among the three that are present, all show elevated A/S ratios, and two of them are also in our top ten list (GO:0007608 sensory perception of smell and GO:0007565 female pregnancy). When GO terms were mapped to a small set of high level terms according to Gene Ontology Annotation [52] (GOA slim), the biological process category with the most relaxed selective constraint was 'response to stimulus', which has a significantly higher A/S ratio of 0.33 compared with 'multicellular organismal development', 'transport', 'macromolecule metabolic process', and 'cell differentiation' (Figure 6a). In terms of molecular function, the least variable groups are 'protein transporter activity' and 'motor activity', and the opposite groups are 'receptor activity' and 'isomerase activity' (Figure 6b).

**Table 1:** GO biological process categories with the highest and lowest SNP A/S ratios

GO accession	A/S ratio	Number of proteins	GO description
GO:0007608	0.72	298	Sensory perception of smell
GO:0050896	0.54	403	Response to stimulus
GO:0007565	0.48	43	Female pregnancy
GO:0006298	0.47	29	Mismatch repair
GO:0031424	0.46	22	Keratinization
GO:0007186	0.43	600	G-protein coupled receptor protein signaling pathway
GO:0007131	0.42	20	Meiotic recombination
GO:0008033	0.40	26	tRNA processing
GO:0045087	0.39	57	Innate immune response
GO:0006633	0.37	20	Fatty acid biosynthetic process
			
GO:0006986	0.14	40	Response to unfolded protein
GO:0006445	0.14	26	Regulation of translation
GO:0006096	0.14	37	Glycolysis
GO:0007420	0.13	25	Brain development
GO:0006334	0.13	38	Nucleosome assembly
GO:0006816	0.12	61	Calcium ion transport
GO:0007411	0.12	20	Axon guidance
GO:0006333	0.10	22	Chromatin assembly or disassembly
GO:0000398	0.09	62	Nuclear mRNA splicing, via spliceosome
GO:0007205	0.07	21	Protein kinase C activation

Top part: ten GO categories with the highest A/S ratios. Bottom part: ten GO categories with the lowest A/S ratios.

We also sought to quantify the selective pressure on protein families. Of the 13,686 proteins in our data set, 10,629 can be assigned to at least one Pfam [53] family using the HMMER program. Among the 190 Pfam families that have at least 20 members, the families with the lowest A/S ratios include protein kinase C-terminal domain family (PF00433) and core histones (PF00125); on the high end there are mammalian taste receptors (PF05296), the rhodopsin family (PF00001), and glutathione S-transferases (PF02798 and PF00043) (Table 2). We took a closer look at the G protein-coupled receptor (GPCR) family. GPCRs comprise a large protein family of seven transmembrane receptors that play important roles in sensing environmental signals. They are the targets of more than 40% of all modern drugs. There are five Pfam GPCR families that have more than 20 proteins in our data set. Mammalian taste receptor proteins (PF05296) and rhodopsin family (PF00001) are among the most variable protein families, with an A/S ratio of 0.49. The other three (PF00002 secretin family, PF00003 metabotropic glutamate family, and PF01461 7TM chemoreceptor) have A/S ratios of around 0.25, similar to the overall A/S ratio of 0.28 in our entire dataset. There are 558 proteins that belong to the rhodopsin family, including 286 olfactory receptors. The elevated A/S ratio in the family can be largely attributed to olfactory receptors (A/S = 0.73): the non-olfactory receptors in this family have an A/S ratio of 0.30. Therefore, it appears that among GPCRs, only olfactory and taste receptors have extraordinarily high variations, while other proteins behave like average human proteins.

**Table 2:** Pfam families with the highest and lowest SNP A/S ratios

Pfam accession	A/S ratio	Number of proteins	Pfam description
PF05296	0.49	55	Mammalian taste receptor protein (TAS2R)
PF00001	0.49	558	7 transmembrane receptor (rhodopsin family)
PF02798	0.47	20	Glutathione S-transferase, amino-terminal domain
PF00043	0.46	24	Glutathione S-transferase, carboxy-terminal domain
PF01454	0.45	24	MAGE family
PF09723	0.44	42	Putative regulatory protein (CxxC_CxxC_SSSS)
PF02023	0.44	39	SCAN domain
PF00059	0.43	58	Lectin C-type domain
PF07859	0.42	21	alpha/beta Hydrolase fold
PF00048	0.40	23	Small cytokines (intecrine/chemokine), interleukin-8 like
			
PF00105	0.14	38	Zinc finger, C4 type (two domains)
PF00536	0.14	68	SAM domain (Sterile alpha motif)
PF07649	0.13	45	C1-like domain
PF00125	0.13	25	Core histone H2A/H2B/H3/H4
PF00535	0.13	27	Glycosyl transferase family 2
PF01437	0.13	31	Plexin repeat
PF00335	0.13	23	Tetraspanin family
PF00350	0.12	28	Dynamin family
PF07707	0.11	36	BTB And C-terminal Kelch
PF00433	0.09	33	Protein kinase C terminal domain

Top part: ten families with the highest A/S ratios. Bottom part: ten families with the lowest A/S ratios.

### Selective pressure on disease-related proteins

**Figure 7:**
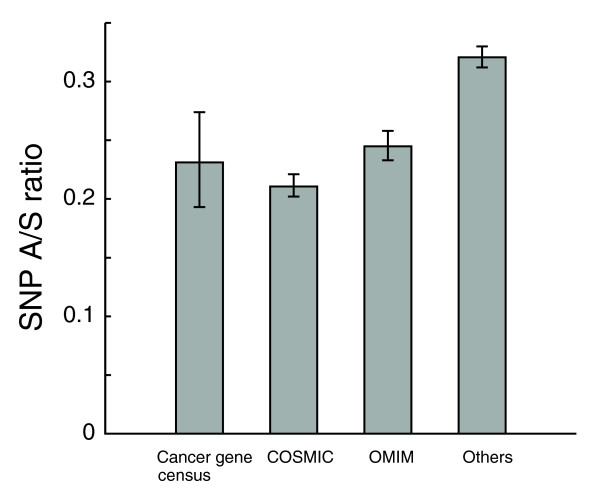
Disease-related genes are under stronger selective pressure. Disease related genes were obtained from CGC (243 genes), COSMIC (3,103 genes), and OMIM (2,334 genes) databases. The SNP A/S ratio was calculated for each group. The 95th percentile confidence intervals from bootstrap resampling (shown as error bars) are [0.19, 0.27] for CGC, [0.20, 0.22] for COSMIC, [0.23, 0.26] for OMIM, and [0.31, 0.33] for others.

Knowledge about the degree of selection for disease-related genes can help us understand the etiology of human diseases. An early study found that human disease genes evolve faster at both synonymous and non-synonymous sites than non-disease genes, and Ka/Ks ratios of disease genes are 24% higher [54]. Although the elevated Ks has subsequently been confirmed by others, later studies reported no difference in Ka/Ks between disease genes and non-disease genes [55] or lower Ka for disease genes [56]. It has also been shown that significant differences exist between the Ka/Ks ratio for different pathophysiological classes: genes related to neurological diseases evolve much slower than those associated with immune, hematological and pulmonary diseases [55]. We investigated the SNP distribution of human disease genes using two cancer-related gene collections (243 genes from Cancer Gene Census (CGC) [57], and 3,103 genes from the Catalogue of Somatic Mutations in Cancer (COSMIC) [58]) and the catalog of heritable human disease genes from Online Mendelian Inheritance in Man (OMIM; 2,334 genes) [27]. These three data sets represent 4,649 unique human genes, and 139 genes are common to all three sets. Our analysis of the SNP data shows that disease related genes indeed have a higher synonymous SNP density (OMIM, 5.14; COSMIC, 4.41; CGC, 4.73; non-disease, 4.19, per 1,000 synonymous sites). However, the numbers of non-synonymous SNPs per site for disease genes are lower than that for non-disease genes, resulting in significantly lower A/S ratios in disease genes (*p*-value < 1e-04; Figure 7). The difference between our analysis and some previous studies could be explained by two factors. First, our data sets are substantially bigger than what were used in previous studies. For example, the Smith and Eyre-Walker study [54] analyzed only 392 genes in the disease set and 2,038 genes in the non-disease set, and the Huang *et al*. study [55] included 1,178 human disease genes. The other possibility is that the evolution of disease-related genes has different patterns in the human lineage, leading to the difference in SNP A/S ratios and Ka/Ks ratios from human-rodent alignments. It has also been suggested that when non-disease genes are partitioned into housekeeping genes and others, the evolutionary rates of disease genes lie between them [59]. This is consistent with our data: the SNP A/S ratio for OMIM is 0.24, indeed higher than housekeeping genes (genes with the broadest expression patterns, A/S = 0.19; Figure 2c). Moreover, when controlling for expression breadth (so that different groups have the same distribution of expression breadth, and thus the same proportion of housekeeping genes), non-disease genes still showed significantly higher A/S ratios than genes in the OMIM and COSMIC sets, while the confidence interval of A/S ratios for genes in the CGC set slightly overlapped with that for non-disease genes (Figure S2c in Additional data file 1), mostly due to large variance in the CGC set resulting from a smaller number of genes.

### Selective constraints and protein-protein interaction

**Figure 8:**
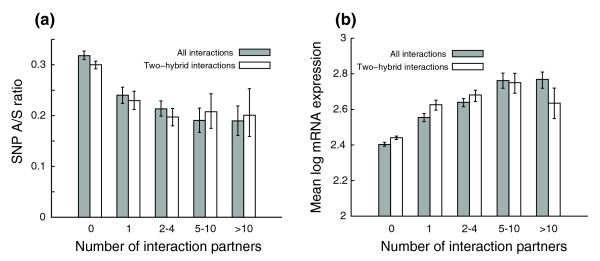
Selective pressures on connectivity in protein-protein interaction networks. Error bars represent 95th percentile confidence intervals from bootstrap resampling. **(a) **Proteins with more interaction partners appear to have lower A/S ratios (gray bars); however, for yeast two-hybrid interactions, the differences are less significant for proteins with at least one interaction partner (white bars). **(b) **Proteins with more interaction partners tend to have higher mRNA expression levels (gray bars). This could result from experimental bias: for yeast two-hybrid interactions, the differences are not significant for proteins with at least one interaction partner (white bars).

It is still debatable whether there is any correlation between protein-protein interaction and selective pressure. Most studies so far have been based on data from the budding yeast *Saccharomyces cerevisiae*. After an initial report that yeast proteins with more interaction partners evolve slowly [60], several studies suggested that the correlation is dependent on interaction data sets [61], or that it may be a secondary effect due to protein abundance [62]. The latest and most conclusive study in yeast suggested that there is no correlation between connectivity and evolutionary rate in a higher quality literature curated interaction data set, while negative correlations observed in some high-throughput data sets even after controlling for expression could be artifacts of the data sets [63]. We obtained human protein-protein interaction data from the IntAct database [64] and examined how SNP A/S ratios are correlated with the connectivity of proteins in the protein-protein interaction network. When all types of interactions were included, proteins with more than five interaction partners appear to have significantly lower A/S ratios than proteins with no more than one partner (Figure 8a, gray bars). We also noticed that proteins with more interaction partners tend to have higher mRNA expression (Figure 8b, gray bars). The Kendall's rank correlation between connectivity and the SNP A/S ratio was -0.131 (*p*-value < 1e-04), and it dropped to -0.106 (*p*-value < 1e-04) after controlling for both mean expression level and expression breadth. The correlation between protein abundance and high connectivity in the interaction network could be either a real biological phenomenon or experimental bias; for example, mass spectrometry-based protein complex pulldown experiments are more likely to identify interaction partners for abundant proteins. When we included only yeast two-hybrid interactions in our analysis, which are supposedly less biased with respect to intrinsic expression levels, the correlation between connectivity and abundance largely disappeared, except for the proteins with no interaction partners in the database (Figure 8b, white bars); at the same time, the difference in A/S ratios between proteins with only one partner and those with more than one became smaller and lost statistical significance in some cases according to bootstrap analysis (Figure 8a, white bars). For yeast two-hybrid interactions only, the correlation between connectivity and the SNP A/S ratio was -0.100, and it dropped only slightly to -0.090 (*p*-value = 0.007) after controlling for expression. Nevertheless, the partial correlations were still statistically significant in both the yeast two-hybrid interaction set and the all interaction set. Our analysis supports the idea that the correlation between evolutionary rate and connectivity in the interaction network can, in part, be explained by protein abundance and that some of the correlation may result from experimental bias. Similar to all the conflicting studies in yeast, it is likely that this result is inconclusive and may vary from data set to data set.

### SNP A/S ratio and splicing

**Figure 9:**
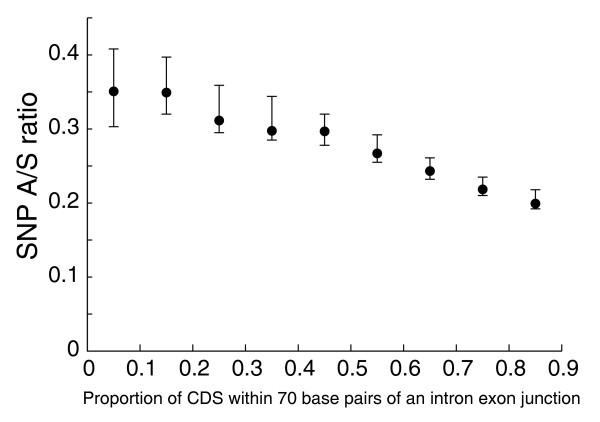
The SNP A/S ratio negatively correlates with the proportion of coding sequence (CDS) within 70 bp of an exon-intron junction. Genes were grouped into bins of nine equal intervals according to the proportion of sequence within 70 bp of an exon-intron junction, and the SNP A/S ratio was obtained for each bin. Error bars represent 95th percentile confidence intervals from bootstrap resampling.

A recent study suggested that protein evolution is strongly affected by mRNA splicing, in addition to the biology of the protein [65]. Based on Ka and Ka/Ks from the human-mouse comparison, it was reported that the proportion of sequence near intron-exon boundaries is a strong predictor of evolutionary rates in human, in part due to splice enhancers located close to intron-exon junctions. We were able to confirm this result using SNP data. Codons within 70 bp of the intron-exon boundaries have a SNP A/S ratio of 0.22, significantly lower than 0.30 for codons that are far from the junction. At the protein level, proteins with more than 80% of the sequences within 70 bp of the boundaries have an SNP A/S ratio of 0.20, much lower than those with only 5% close to the boundaries (Figure 9). The Kendall's rank correlation between the proportion of sequence near intron-exon boundaries and SNP A/S ratio is -0.163, comparable to the correlation between mean mRNA expression levels and the SNP A/S ratios. After controlling for expression breadth, the correlation remained significant (τ = -0.147, *p*-value < 1e-04).

## Discussion

### Measuring selective constraints with SNP A/S ratios

The average SNP density in our data set is about 2 SNPs per 1,000 nucleotides. Although we have limited our analysis to proteins with at least one validated coding SNP, many proteins in our set still have no non-synonymous SNPs or synonymous SNPs. Therefore, it is neither practical nor reliable to measure the selective constraints on individual proteins using the SNP A/S ratio. Nevertheless, we have demonstrated that when a group of proteins (or residues) are measured together, the measure can be quite robust and often in good agreement with Ka/Ks for divergence. For example, the SNP A/S ratio for proteins with paralogs in our data set is 0.47, very close to the Ka/Ks ratio of 0.45 for duplicated mammalian genes reported earlier [25]. Although this may not seem surprising given that it is an expected prediction from the neutral theory of evolution, our analysis showed that the SNP A/S ratio can indeed be a practical and robust measure given the current volume of SNP data in public databases.

There are two unique advantages of using SNP A/S ratios to measure selective constraints. First, for human-specific genes and genes whose ortholog relationships can not be determined reliably, inter-species Ka/Ks ratios can not be estimated. For example, among the 13,686 genes in our data set, human-mouse Ka/Ks ratios for 3,937 genes are not available through Ensemble. In those cases, SNP A/S ratios provide an alternative way of measuring selective pressure by the same evolutionary principle. Second, the SNP A/S ratio is a direct measure of the selective constraints specific to the human lineage, which can not be obtained from Ka/Ks for species divergence.

In comparison with other simpler measures used in some earlier SNP studies (for example, [38]), such as density of synonymous and non-synonymous SNPs or the fraction of SNPs in different protein classes, the SNP A/S ratio offers several advantages: its interpretation is clearer from evolutionary theories; it is not subject to data selection bias arising from the popularity of genes in sequencing efforts, which could be a problem when using SNP density; and it is normalized by the number of synonymous and non-synonymous sites, so the numbers are comparable across different protein classes or different studies.

### Correlation between SNP distribution and structural/functional constraints

To address the issue of determinants of molecular evolution, many studies have been published examining the correlation between variables that characterize the evolution, expression and function of genes. However, as noted in the Results section, there are many controversies among those reports. It is clear that the validity and significance of correlations depend on many factors, including feature variables, data sets, and the correlation measure. Here, we provide the first large-scale study of SNP A/S ratios, and reported correlations between the ratio and a number of variables; not surprisingly, some are not in agreement with previous studies. Although further studies are still needed to draw definitive conclusions about the major determinants, we agree with many others that it is likely that many of these variables correlate with each other in some way, and some of them are secondary effects rather than primary determinants [66]. Several statistical methods, such as partial correlation and principle component regression, have been used to attempt to dissect these complex and rich connections; yet it remains an important open challenge in molecular evolution.

## Conclusion

Molecular evolution is a dynamic process, with strong implications for both differences between species and variations within a given species. SNPs in the human genome perhaps capture a glimpse of this dynamic process and, therefore, could offer key insights missed by conventional cross-species comparisons. Here, we first established the SNP A/S ratio as a reliable metric for studying the selective constraints for molecular evolution, and then used this metric to systematically investigate a large number of protein features that contribute to differences in molecular evolution rate. Our study provided the first such large-scale survey based on SNP A/S ratios, leading to novel insights into features that have not been examined before and clarification of findings that were contradictory in the literature.

## Materials and methods

### Source of sequences and SNP data

We first filtered the human entries in the NCBI's Entrez Gene database [27] by the presence of RefSeq [27] records, and limited our analysis to genes that have at least one validated coding SNP according to dbSNP [6] build 127. For each gene, we chose one transcript (and its corresponding protein product) by selecting the transcript with the most validated coding SNPs, most advanced RefSeq annotation status, and the longest sequence. We also discarded proteins that are longer than 5,000 residues or shorter than 25 residues. Our final data set has 13,686 proteins with 45,538 coding SNPs.

### mRNA expression data

mRNA expression data were obtained from Gene Expression Atlas [24,67]. Only normal adult samples were included in the analysis. Samples were sorted into 54 non-redundant tissue types. The expression level of each probe set in a given tissue was calculated as the mean of log (base 10) MAS5 signal intensities of all samples in that tissue. The 'mean expression level' of a probe set was defined as the mean across all tissues, while 'peak expression level' was defined as the maximum among all tissues. The tissue specificity of a probe set was defined as the heterogeneity of its expression level across all tissues. It was calculated according to [68] as:

$$\frac{\sum_{j=1}^{n}\left(1-\frac{\log S_{j}}{\log S_{\max}}\right)}{n-1}$$

where *n* = 54 is the number of human tissues examined here, S_*j*_ is the expression level in each tissue, and *S*_max_ is the highest expression level of the probe set across all tissues. When a gene has multiple probe sets, its expression levels and tissue specificity were represented by the probe set with the highest mean expression level.

Affymetrix present/absent calls were used to calculate the breadth of the expression. A probe set was considered 'present' in a tissue if it had 'present' calls in no less than half of the samples in that tissue, and the expression breadth of a probe set was defined as the number of tissues in which the probe set was 'present'. When a gene has multiple probe sets, its expression breadth was represented by the probe set with the highest value of breadth.

### Structural and functional features

Ka/Ks ratios from human-mouse alignments were downloaded from Ensembl [13]. The information about human paralogs was extracted from HomoloGene database [27,28] release 57; the existence of paralogs is indicated by the presence of other human proteins in the same homology group. To investigate the degree of conservation throughout the evolutionary history, that is, the age of a protein, we performed BLAST searches [15] of each protein sequence against NCBI's RefSeq database, and collected all hits with an e-value < 1e-10 and a protein length difference smaller than 30% as potential homologs. The query protein was then classified into one of the seven age groups (human, primate, mammal, vertebrate, animal, eukaryote, or universal) according to its most ancient homolog. The use of different e-values and length difference cutoffs did not change the results qualitatively. We also obtained the conservation score for each residue in all proteins in our data set by running PSI-BLAST [15] against NCBI's nr database (parameters: '-j 3 -h 5e-3 -F F') and taking the values in the 'information per position' (a measure of alignment entropy) column from the ASCII format of PSI-BLAST profiles.

We obtained protein structure features by the following computational methods using their default parameters: two-state (exposed or buried) solvent accessibility by PROFacc [40], signal peptides by SignalP version 3.0 [49], transmembrane helices by TMHMM 2.0 [50], secondary structures by PSIPRED [41], sequence complexity by SEG [46], and natively disordered proteins by Disopred2 [43]. Disopred2 predictions were subsequently filtered to retain only the disordered regions longer than 30 residues.

GO annotations [32] were extracted from the GenBank record of each gene, and the GO terms were subsequently mapped to a selection of high-level terms according to GOA slim [52,69]. For cellular component annotations, the GOA slim terms were further collapsed into four categories (extracellular region, nucleus, cytoplasm, and membrane) where appropriate. Proteins assigned to more than one of these four categories were excluded from the analysis of subcellular localization by GO annotation. Subcellular localization prediction for non-membrane proteins (that is, no transmembrane helix predictions from TMHMM) were also obtained by using LOCtree [51]. To assign proteins to protein families, we ran hmmpfam from the HMMER package (version 2.3) against Pfam_ls [53] models (release 21.0) and obtained Pfam family hits with an e-value < 0.01. Numbers of protein-protein interaction partners were obtained from the IntAct database [64,70] (28 September 2007 release). We also obtained disease related genes from CGC (243 genes) [57,71], COSMIC [58,72] (3,103 genes), and OMIM databases [27,73] on 28 September 2007. For OMIM, only those genes with the 'confirmed' status (2,334 genes) were included in the analysis.

Sequences within 70 bp of an exon-intron junction were collected in the same way as described in [65]. Briefly, transcripts with less than three exons were excluded from the analysis. All internal exons were trimmed so that the first base was the first base of the first complete codon, and the last base the last of the final complete codon. The first and last codons were then removed from each exon, and remaining codons within 70 bp of the intron-exon boundary were defined as sequences close to the boundary.

The features we considered can also be divided into two classes: protein-level features and residue-level features. A protein-level feature describes the property of an entire protein, for example, whether a protein has a transmembrane helix or not; in contrast, a residue-level feature describes the property of a subset of residues within a protein, for example, whether a residue resides in the transmembrane helix or not.

### Data analysis

The SNP A/S ratio, also known as the Ka/Ks ratio for polymorphism, is defined as the ratio of the number of non-synonymous SNPs per non-synonymous site to the number of synonymous SNPs per synonymous site. The numbers of synonymous sites and non-synonymous sites were calculated using the method of Miyata and Yasunaga [74]. The ratio for a set of proteins (or residues) was calculated by summing the number of SNPs and the number of sites to obtain A and S for the concatenated set before taking the ratio.

We performed bootstrap re-sampling analysis to assess the statistical significance of the differences observed in A/S ratios between different groups. We obtained 10,000 bootstrap replicates by re-sampling with replacement from the original data set. A/S ratios for different groups were calculated for each replicate, and confidence intervals and *p*-values of the differences were obtained from those 10,000 sets of A/S ratios. For protein-level feature groups, re-samplings were performed within each group, so that those re-sampled data sets had the same number of proteins in each group as the original data set had. For residue-level feature groups, the re-sampled data sets were constructed by re-sampling the 13,686 proteins in our entire data set.

Monte Carlo samplings were used to assess the differences in A/S ratios between groups when controlling for expression parameters. Briefly, the distribution of expression breadth (or expression level) for the group with the smallest number of genes was set as the target distribution; genes in all other groups were then sampled without replacement using Monte Carlo simulation so that all groups had the same distribution of the expression breadth (or level). The effectiveness of Monte Carlo samplings was confirmed by Kolmogorov-Smirnov tests. An example is shown in Figure S1 in Additional data file 1. For each group, 100 samplings were performed, and the A/S ratio for the group was taken as the mean of A/S ratios from the 100 Monte Carlo samples.

Since the average SNP density in our data set is about 2 SNPs per 1,000 nucleotides, and many proteins in our data set have either no non-synonymous SNPs or no synonymous SNPs, it is not possible to reliably calculate the correlation between the SNP A/S ratio and other continuous variables using each protein as a data point. We chose to randomly group every six proteins together as a data point so that, on average, each data point had roughly the same number of SNPs as the reported number of single-nucleotide substitutions (1.23%) between the human and chimpanzee genomes [12]. Non-parametric Kendall's τ rank correlation coefficients [14] and two-tailed *p*-values were used throughout the study. When controlling for expression parameters, Kendall's partial correlation between x and y controlling for z was calculated as:

$$\tau_{xy.z}=\frac{\tau_{xy}-\tau_{xz}\tau_{yz}}{\sqrt{\left(1-\tau_{xz}^{2})(1-\tau_{yz}^{2}\right)}}$$

The random grouping was performed 100 times. The correlation and partial correlation coefficients were computed from these 100 samples, and the medians of those 100 sets of coefficients were reported.

## Abbreviations

CGC, Cancer Gene Census; COSMIC, Catalogue of Somatic Mutations in Cancer; GO, Gene Ontology; GPCR, G protein-coupled receptor; Ka, non-synonymous substitutions per non-synonymous site; Ks, synonymous substitutions per synonymous site; OMIM, Online Mendelian Inheritance in Man; SNP, single nucleotide polymorphism.

## Authors' contributions

ZZ and JL designed the study. JL and YZ collected the data and performed the data analysis. XL participated in the statistical analysis. JL and ZZ drafted the manuscript. All authors read and approved the final manuscript.

## Additional data files

The following additional data are available. Additional data file 1 includes Table S1 and Figures S1-S3.

## Supplementary Material

Additional data file 1 Table S1 presents correlation and partial correlations between SNP A/S ratios and expression parameters. Figure S1 shows an example of using Monte Carlo sampling to get the same distributions of expression breadth for different groups of proteins. Figure S2 and S3 demonstrate that for most variables in our study, the differences in A/S ratios between groups do not change qualitatively after controlling for expression breadth. Click here for file
