# Supplementary material for: Natural selection of protein structural and functional properties: a single nucleotide polymorphism perspective
Source: Genome Biol. 2008 Apr 8;9(4):R69. doi: 10.1186/gb-2008-9-4-r69 (PMC2643940; doi:10.1186/gb-2008-9-4-r69)
Supplement: Additional data file 1 — Table S1 presents correlation and partial correlations between SNP A/S ratios and expression parameters. Figure S1 shows an example of using Monte Carlo sampling to get the same distributions of expression breadth for different groups of proteins. Figure S2 and S3 demonstrate that for most variables in our study, the differences in A/S ratios between groups do not change qualitatively after controlling for expression breadth. [file gb-2008-9-4-r69-S1.pdf]

Table S1 – Kendall's rank correlation and partial correlations between SNP A/S ratios and expression parameters

X	Y	Z	τ_{xy}	$\tau_{xy.z}$	$P_{xy.z}$	τ_{xz}	$\tau_{xz.y}$	$P_{xz.y}$	τ_{yz}
A/S	Breadth	Mean	-0.213	-0.096	1e-04	-0.178	-0.064	1e-04	0.523
A/S	Breadth	Peak	-0.213	-0.139	1e-04	-0.160	-0.070	1e-04	0.428
A/S	Breadth	Specificity	-0.213	-0.187	1e-04	0.047	0.0084	0.596	-0.192
A/S	Mean	Peak	-0.178	-0.0679	1e-04	-0.160	-0.044	0.005	0.666
A/S	Mean	Specificity	-0.178	-0.154	1e-04	0.047	0.0107	0.499	-0.248
A/S	Peak	Specificity	-0.160	-0.164	2e-04	0.047	0.0555	4e-04	0.083

τ_{xy}: Kendall's rank correlation between variable X (A/S ratio) and variable Y (e.g., expression breadth)
$\tau_{xy.z}$: Kendall's partial rank correlation between variable X (A/S ratio) and variable Y (e.g., expression breadth) when controlling for variable Z (e.g., mean expression level).
$P_{xy.z}$: p-value associated with $\tau_{xy.z}$

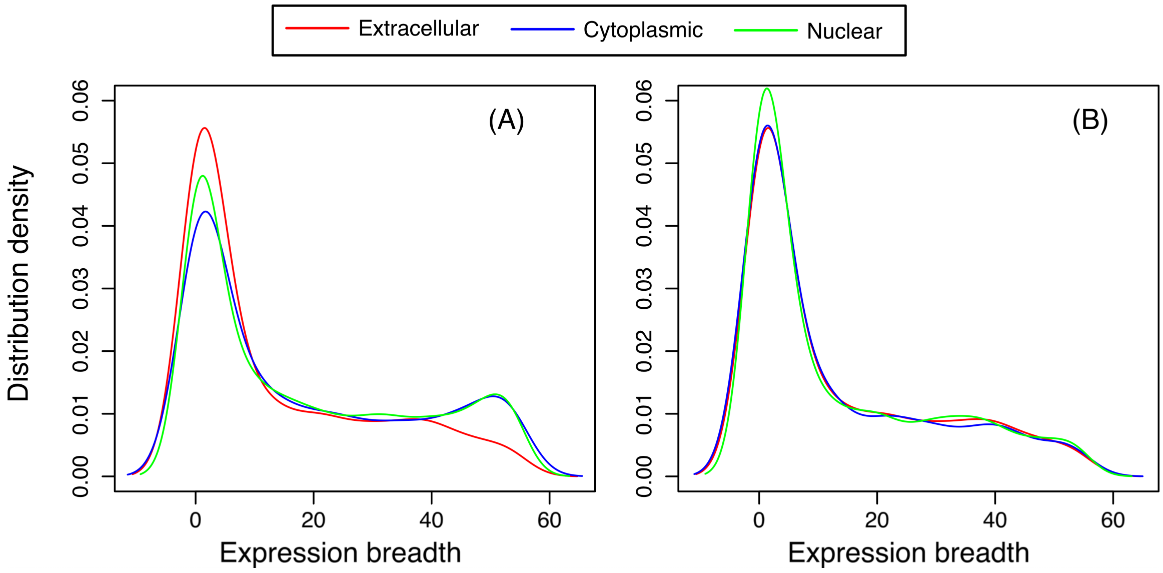

Figure S1 - An example of using Monte Carlo sampling to get the same distributions of expression breadth for different group of proteins. (A) Proteins with different subcellular localizations have different distributions of expression breadth. P-values from Kolmogorov-Smirnov tests are 6.9e-08 for extracellular proteins vs. cytoplasmic proteins and 1.3e-11 for extracellular proteins vs. nuclear proteins. (B) After Monte Carlo sampling, all groups have the same distribution. P-values from Kolmogorov-Smirnov tests are 0.888 for extracellular proteins vs. cytoplasmic proteins and 1 for extracellular proteins vs. nuclear proteins, indicating the three distributions are similar.

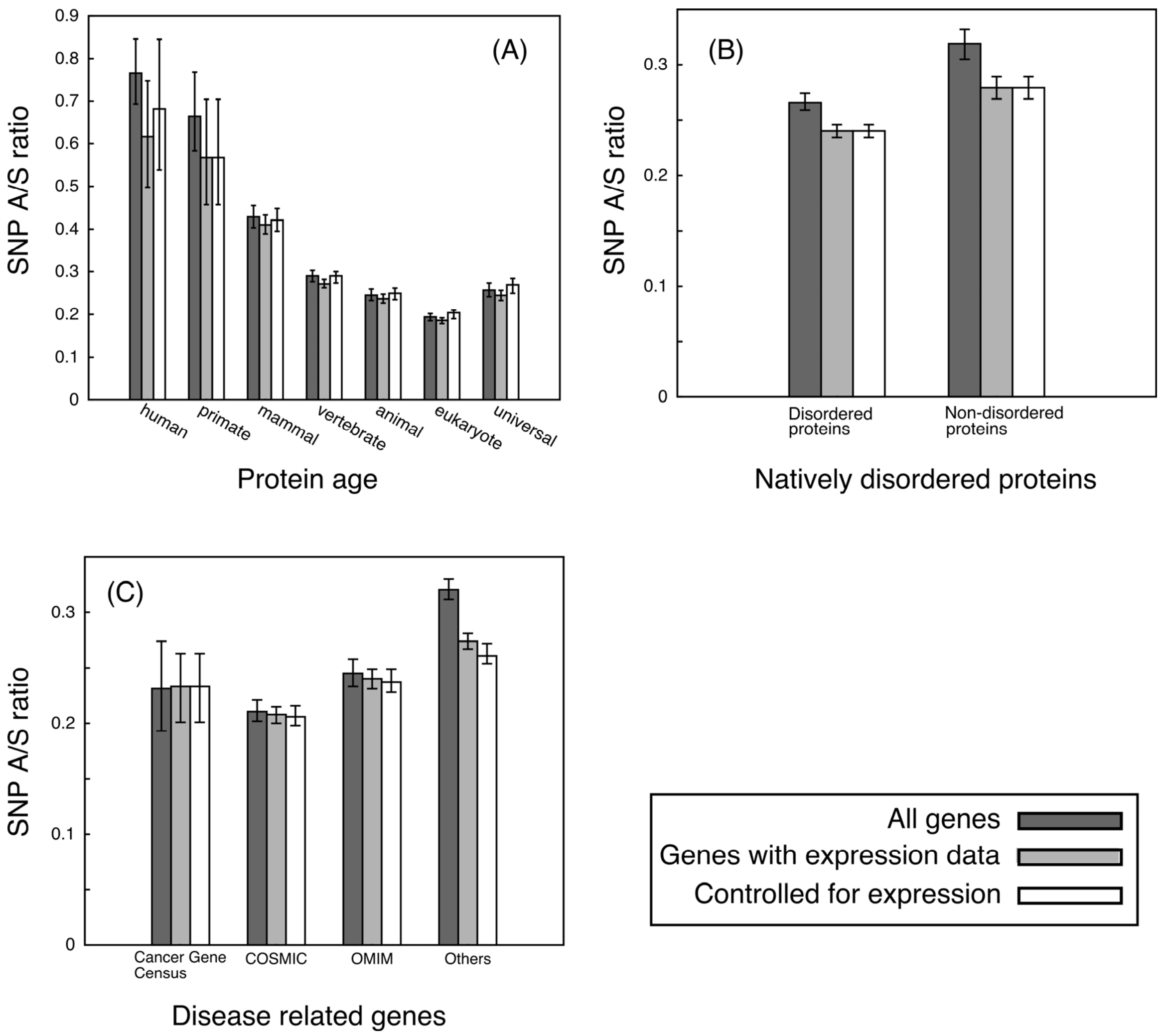

Figure S2 – For several variables we studied, the differences in A/S ratios between groups do not change qualitatively after controlling for expression breadth. Dark gray bars: all genes in our data set; light gray bars: genes with available mRNA expression data from a microarray experiment; white bars: genes remained in each group after Monte Carlo samplings (i.e., after controlling for expression). (A) Protein age; (B) Natively disordered proteins; (C) Disease-related genes.

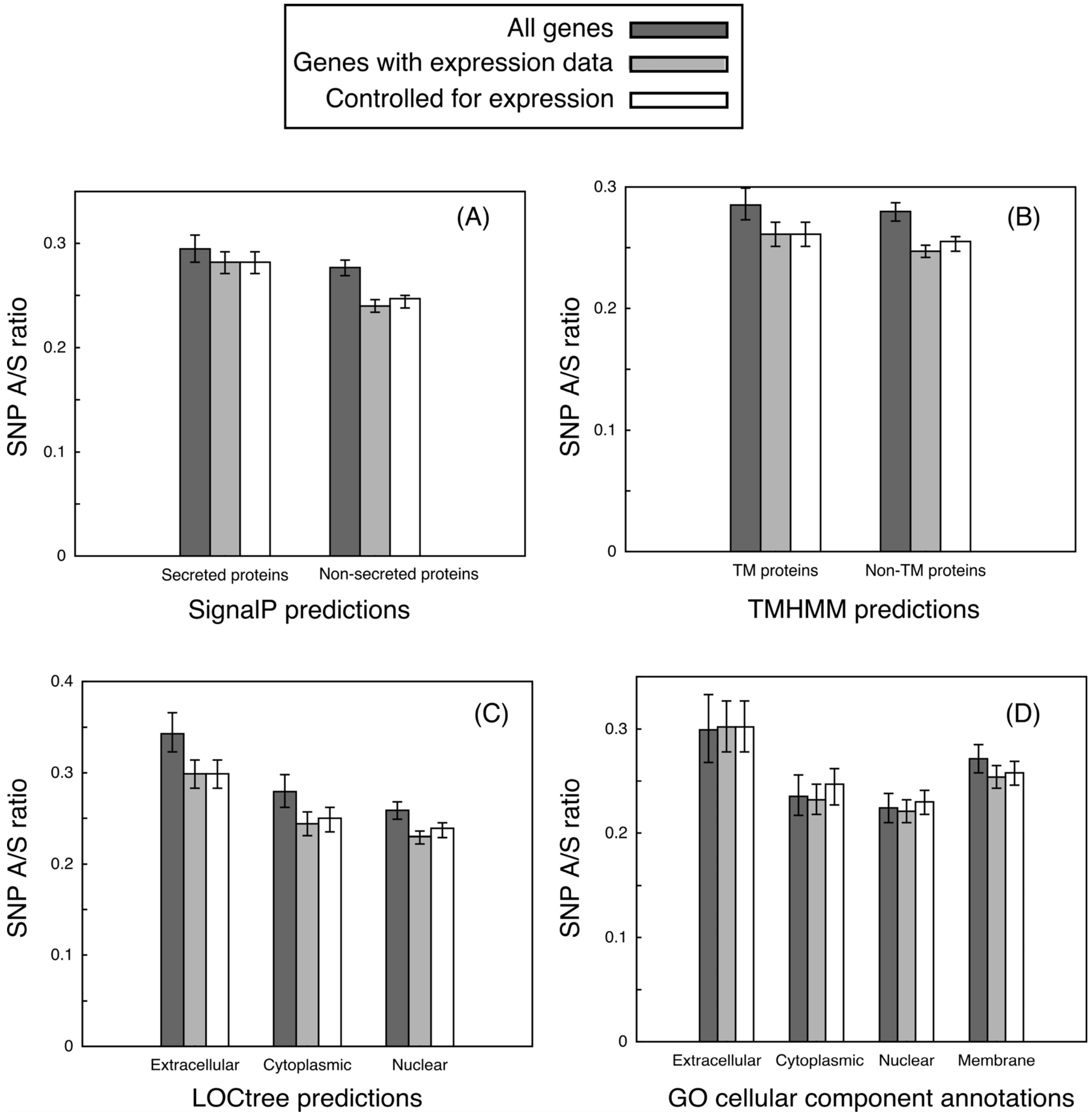

Figure S3 – SNP A/S ratios and subcellular localization, controlling for expression breadth. Dark gray bars: all genes in our data set; light gray bars: genes with available mRNA expression data from a microarray experiment; white bars: genes remained in each group after Monte Carlo samplings (i.e., after controlling for expression). (A) After taking the subset of proteins that have available mRNA expression data, the difference between secreted and non-secreted proteins become significant (light gray bars and white bars); the results do not change qualitatively for (B) transmembrane and non-transmembrane proteins according to TMHMM predictions, (C) subcellular localization predictions from LOCtree, (D) Gene Ontology cellular component annotations.
